# Epidemic pattern of hand-foot-and-mouth disease in Xi’an, China from 2008 through 2015

**DOI:** 10.1186/s12879-018-3624-5

**Published:** 2019-01-07

**Authors:** JiFeng Liu, XiaoMei Xiang, ZhongShu Pu, Yong Long, Dan Xiao, WeiLu Zhang, Qian Li, XiTong Li, SiYao Li, ZhongJun Shao, XiaoLi Yang, YongMin Xiong

**Affiliations:** 1 Institute of Endemic Diseases of School of Public Health, Health Science Center, Key Laboratory of Trace Elements and Endemic Diseases of National Health and Family Planning Commission, Xi’an Jiaotong University, No.76, YanTa West RD, Xi’an, 710061 Shaanxi China; 2 Xi’an Center for Maternal and Child Care Service, Fourth Military Medical University, No.73, Xi’an West Street, Xi’an, 710002 Shaanxi China; 3 Department of Epidemiology, School of Public Health, Fourth Military Medical University, No. 17, Changle West RD, Xi’an, 710032 Shaanxi China; 4 Center of Disease Control and Prevention of Xi’an, Shaanxi Province, China; No,599 Xiying Rd, Xi’an 710054, Xi’an, 710061 Shaanxi China

**Keywords:** Hand-foot-and-mouth disease, Epidemic, Distribution, Serotype

## Abstract

**Background:**

Hand, foot and mouth disease (HFMD) is an infectious disease caused by enteroviruses that has a severely impair for those high incidence countries such as China.The current study aimed to investigate the epidemic pattern of HFMD by time and region in Northwestern China.

**Methods:**

All reported HFMD cases from 2008 to 2015 were collected from local Disease Control and Prevention.The HFMD was diagnosed in accordance with the guidebook provided by the National Health and Family Planning Commission of the People’s Republic of China.

**Results:**

A total of 154,869 cases of probable HFMD were reported. The overall incidence of HFMD has been increased from 91.68 per 100/000 in 2008 to 335.64 per 100/000 in 2015.The case mortality is decreased from 0.014 per100/000 to 0.011 per 100/000 during the time period. Most HFMD (93.82%) occurred in children younger than 5 years. The seasonal peak of HFMD infections occurred in April–July and September–November and Central regions of Xi’an city were the major locations of the clusters (incidence rate 245.75/100,000; relative risk 1.19, *P* < 0.01). EVA71 was the predominant enterovirus serotype, accounting for 50.0% of all reported HFMD cases since 2011.The most susceptible group infected by HFMD was children younger than 5 years, especially boys.

**Conclusions:**

Incidence of HFMD has been increasing in the past few years, however, the case fatality is decreasing. Season and region shall be considered as influence factors in the prevention of HFMD.

## Background

Hand-foot-and-mouth disease (HFMD) is a viral infectious disease caused by human enteroviruses. It is typically characterized by fever, sore throat, general malaise, and vesicular rash on the hands and feet, as well as exanthema on oral mucosa and tongue [1]. Two serotypes of enteroviruses, A71 (EVA71) and Coxsackie virus A16 (CVA16), regarded as the major causes for the repeated national HFMD outbreaks in China [2].

To achieve better control and prevention of HFMD, many studies have explored the epidemiological distribution pattern of HFMD in some areas of China [2–4], including the provinces of Guangdong [5, 6], Hainan [7], Guangxi [8], and Shaanxi [9]. The consensus of these studies was that most HFMD cases were children younger than five years, with boys having the higher incidence rate. However, regions of China have varied in the temporal and spatial distributions of high HFMD incidence rates, and also the predominate serotype.

Xi’an city is the capital of Shaanxi Province, an area that is continuously threatened seriously by HFMD [3]. Xi’an, locates at 33°42′-34°45’ N, 107°40′-109°49′ E, the central of China, with a population size of 8.63 million, and contains 13 counties and districts [10]. The landscape of Xi’an is flat with climate of temperate continental monsoon, which is favorable for the transmission of HFMD. Outbreaks of HFMD occur frequently in Xi’an [11, 12], which has imposed a substantial disease burden for local government. To better control and prevent this disease, a clear epidemiological pattern of this disease is needed. However, until now, no study has systematically analyzed the temporal-spatial distribution of HFMD infections, and the association between different serotypes and severity of disease in Xi’an. Therefore, in the current study, we mainly aimed to explore the distribution pattern of HFMD by time period and region, and to identify the major enterovirus serotypes causing this disease.

## Methods

### Data collection

The surveillance data of HFMD in Xi’an city from 2008 to 2015 were obtained from Xi’an Center for Disease Control and Prevention (CDC). Disease-related demographic information, such as gender, occupation, age, date of diagnosis, and disease severity (mild or severe) were collected. In China, HFMD is a national class C notifiable communicable disease and all clinics are obliged to report HFMD cases to the local CDC within 24 h. The HFMD web-based surveillance network comprises 105 clinics and local CDCs in 13 counties and districts in Xi’an. The HFMD was diagnosed in accordance with the guidebook provided by the National Health and Family Planning Commission of the People’s Republic of China (version 2008). The CDC laboratory did PCR test through the surveillance program that each district send HFMD samples to city level CDC every month,which include the mild cases,the severe and death case sample. All of the laboratory tested cases were randomly selected by the clinics and district CDC, and these cases composed of our study sample. Most of the HFMD cases are mild thus we could not collect all of the mild cases through the surveillance system, but died and severe cases were less and we tried our best to collect them, and also, not all of the HFMD cases could be included in our study.

### Specimen collection and virological investigations

Throat swabs and urine and fecal samples were collected from outpatients and inpatients in each clinic and local center. Five microliters of viral RNA was extracted from each patient. Reverse transcription and real-time polymerase chain reaction (PCR) was performed to identify the infecting enterovirus using commercial enterovirus detection kits (Da An Gene, Guangzhou, China). The PCR kit we used was three monoplex PCR kits. The enterovirus infection results were classified into four groups in accordance with the manufacturer’s instructions: EVA71-positive, CVA16-positive, pan-enterovirus, or enterovirus-negative. To ensure the reliability of results, all tests were conducted at the Xi’an CDC laboratory, with a biosafety level 2.

### Seasonal variation analysis

The monthly number of HFMD cases was used to calculate the seasonal indices from 2008 through 2015 in Xi’an City. The seasonal index was calculated as:

$$ {S}_k=\frac{x_k}{\overline{x}},\mathrm{where}\ {x}_k=\frac{N_k}{P_k}\ \mathrm{and}\ \overline{x}=\frac{\sum \limits_{i=1}^n{N}_i}{12\times \sum \limits_{i=1}^n{P}_i} $$

The term *S*_*k*_denotes the seasonal index in month *k*, where*k* = 1, 2, ...12. The term *x*_*k*_is the incidence rate in month k; $\overline{x}$is the mean incidence rate during the entire study period. *N*_*k*_ is the number of cases in month k, *P*_*k*_ denotes the population in month k, where k = 1,2,…12. The parameter *N*_*i*_ denotes the number of cases in year i, and *P*_*i*_,means the population in year i, where *i* = 1,2,…8,The parameter *n* denotes the total number of years, which was 8 in this study. If all the seasonal indices in each month were close to 1, this meant that no obvious seasonal fluctuation was detected.

### Secular trend analysis

The annual HFMD incidence rates during 2008–2015 in Xi’an city were calculated and plotted to show the annual fluctuations in HFMD infections. The Cochran-Armitage trend test was employed to examine the temporal trends in annual HFMD incidence during 2008–2015. *Z* > 0 denoted an increasing trend, while *Z* < 0 denoted a declining trend. The trend was considered significant when *P* was < 0.05. The Cochran-Armitage trend test was performed using SAS 9.2 (SAS Institute, USA).

### Spatial distribution analysis

A spatial cluster analysis was used to analyze the spatial autocorrelation association of HFMD incidence based on the locations of study regions. The annual incidence of HFMD in each county or district was mapped for the years 2008 through 2015. Each region was marked with a different color on the county-level digital map.

A spatial cluster analysis of HFMD incidence rate from 2008 through 2015 was conducted to detect the high-risk areas of HFMD in Xi’an City. The relative risk (RR) of HFMD among different regional clusters was calculated to compare the difference in incidence rate among those regions. The most likely cluster was the area with the highest HFMD risk, and the secondary cluster indicated an area with the second-highest HFMD risk. A maximum cluster size of 30% of the study population was specified in the spatial cluster analysis. The spatial cluster analysis was performed using SatScan 7.0.3 (Information Management Services, Boston, MA, USA).

### Enterovirus serotype distribution analysis

The annual proportion of cases infected by the different enterovirus serotypes from 2008 through 2015 was calculated and plotted to show the distribution and variations in etiology of HFMD in Xi’an City. The median age, fatality rate due to HFMD, and proportion of severe cases infected by different enterovirus serotypes was calculated.

### Basic epidemiological and statistical analysis

Descriptive statistics (distributions of age, gender, rates of incidence and mortality rate, fatality rate, and disease severity) were used to describe the epidemiological characteristics of HFMD. Chi-squares test was applied to compare the age and gender distributions of HFMD incidence rate.

## Results

### Demographic incidence trend of HFMD

There were 154,869 cases of probable HFMD infection reported in Xi’an city from 2008 through 2015. The average incidence and mortality rates were 235.01 and 0.041 per 100,000, respectively (Table 1). The average fatalities rate was 0.017%. The highest incidence rate of HFMD was observed in the years 2015 (328.02/100,000), followed by the year 2010 (325.23/100,000) (Table 3). The mortality rate reached peak in the year 2012 (0.071/100,000), and case fatality rate peaked in the year 2009 (0.043%).

**Table 1:** The annual reported HFMD cases, incidence rate, mortality rate, and case fatality rate in Xi’an City, China (2008–2015)^a^

	Cases, n	Incidence/100,000	Mortality/100,000	Case fatalities, %
2008	7065	91.53	0.013	0.014
2009	11,533	149.00	0.065	0.043
2010	25,200	325.23	0.065	0.020
2011	12,374	146.13	0.024	0.016
2012	25,348	298.17	0.071	0.024
2013	19,918	233.09	0.012	0.005
2014	25,257	294.06	0.047	0.016
2015	28,174	328.02	0.035	0.011
Total	154,869	235.01	0.041	0.017

^a^Data reported as number of cases, unless indicated otherwise

**Fig. 1:**
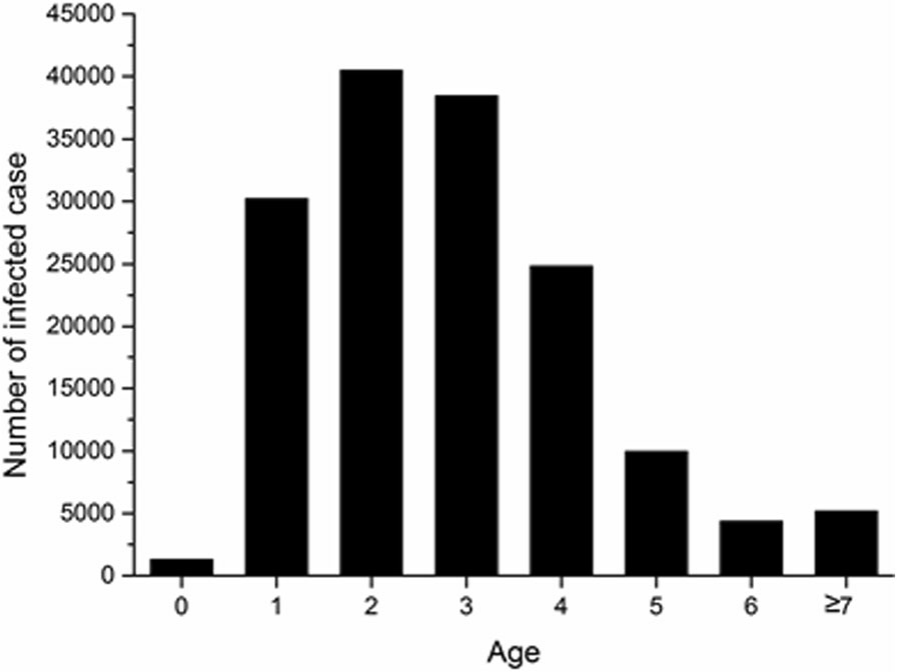
Age distribution of HFMD infections overall during 2008–2015 in Xi’an City, China

The trend in HFMD infections by age group was shown in Fig. 1. The age range of reported cases was 0.1 to 80 years, with a median age of 3.0 years. The ages of most cases of probable HFMD were in the range of 1–4 years, accounting for 86.53% of 134,013 cases. Approximately 93.82% cases were younger than 5 years.

Of the 154,869 probable HFMD cases, 92,193 (59.53%) were males and 62,676 (40.47%) were females (Table 2). The highest incidence rate of HFMD infection were observed in age groups of 1-year (6167.17/100,000), 2-year (5368.42/100,000), and 3-year (5264.54/100,000). Males (273.29/100,000) had higher HFMD incidence rate than females (197.01/100,000) in Xi’an City (χ^2^ = 11.996, *P* = 0.001, data not shown).

**Table 2:** Incidence of HFMD infection per 100,000 by year, gender, and age from birth to 10 years

		Age, y											
		0	1	2	3	4	5	6	7	8	9	10	Total
2008	Male	436.53	1362.39	2380.90	2334.80	1578.39	506.05	273.32	131.29	84.11	64.75	1.98	109.67
	Female	284.19	861.73	1676.18	1628.30	993.70	379.52	179.76	82.51	81.93	56.30	1.19	72.58
	Total	363.30	1122.21	2042.83	1995.85	1297.89	445.35	228.43	109.68	83.15	60.97	1.60	91.68
2009	Male	864.62	1893.24	4127.35	3698.19	1975.15	998.61	384.69	166.30	120.75	59.06	1.87	173.13
	Female	547.43	1271.04	2963.49	2851.10	1498.59	741.71	244.91	121.82	96.57	96.46	1.94	123.69
	Total	712.13	1594.76	3569.03	3291.81	1746.52	875.36	317.62	144.96	110.03	75.53	1.90	149.14
2010	Male	1887.43	7925.33	8221.74	6875.22	3244.56	1364.83	585.62	224.68	191.91	146.11	4.88	378.75
	Female	1353.95	5779.97	5801.57	5005.03	2485.78	967.01	440.81	153.46	137.04	107.38	4.28	268.87
	Total	1630.95	6896.30	7060.84	5978.03	2880.54	1173.96	516.14	190.51	165.58	128.95	4.59	325.40
2011	Male	1256.33	4463.24	4100.00	4236.90	2072.98	908.64	291.42	119.46	93.95	101.70	1.90	172.62
	Female	849.54	3084.01	2855.33	3023.84	1412.37	650.47	302.90	89.48	73.06	99.20	1.49	118.73
	Total	1060.16	3799.31	3501.12	3653.00	1755.03	784.32	296.94	105.03	83.89	100.59	1.70	146.35
2012	Male	3392.07	9606.99	8990.30	8767.84	4431.84	1724.01	735.71	285.68	198.37	167.72	4.05	346.42
	Female	2465.55	7645.50	6724.38	6450.22	3342.04	1351.23	527.40	289.08	153.35	190.89	4.06	247.14
	Total	2952.52	8687.36	7923.16	7674.81	3917.19	1550.19	638.65	287.33	176.43	177.66	4.06	297.98
2013	Male	3924.49	9695.87	5694.52	5831.32	2619.58	1110.64	423.59	130.45	69.86	123.11	2.81	269.20
	Female	2969.96	7827.37	4745.05	4090.87	1877.58	829.00	302.75	96.24	69.37	159.69	2.65	195.09
	Total	3472.81	8822.24	5248.50	5012.31	2269.82	979.52	367.36	113.15	69.61	138.76	2.73	233.09
2014	Male	2273.79	9718.80	7407.49	8532.81	4210.74	1511.03	719.19	250.76	146.28	133.20	2.85	336.56
	Female	1823.08	7941.76	6245.69	6565.63	3166.08	1186.55	677.87	219.63	115.65	156.90	3.07	249.94
	Total	2060.98	8884.03	6873.93	7599.11	3715.69	1358.70	699.91	235.00	130.48	143.59	2.96	294.06
2015	Male	4316.09	12,573.95	7807.17	8861.40	4245.72	1849.00	777.76	245.05	158.68	172.09	4.66	384.51
	Female	3234.81	10,572.90	6691.21	6882.51	2928.86	1309.86	570.57	225.67	95.49	178.61	3.40	284.78
	Total	3800.87	11,632.01	7289.34	7923.10	3620.20	1595.61	680.41	235.18	126.23	174.94	4.04	335.63
Total	Male	2129.00	6908.65	6039.67	6011.85	2988.52	1234.51	518.30	196.49	134.50	116.51	3.14	273.29
	Female	1550.78	5348.04	4621.60	4444.30	2163.41	911.50	397.29	164.48	103.55	123.18	2.78	197.01
	Total	1852.57	6167.17	5368.42	5264.54	2595.02	1081.28	460.94	180.85	119.39	119.44	2.96	236.11

**Table 3:** Incidence of HFMD infection per 100,000 by year and region

	Xincheng	Beilin	Lianhu	Baqiao	Weiyang	Yanta	Yanliang	Lintong	Chang’an	Lantian	Zhouzhi	Hu	Gaoling	Total
2008	82.84	77.00	130.48	107.48	191.89	143.00	39.98	85.12	109.95	33.03	8.21	37.60	127.55	91.68
2009	152.32	86.09	221.47	154.29	335.20	260.88	61.22	63.64	151.81	127.15	45.59	53.27	246.26	149.14
2010	254.85	188.81	334.98	279.83	663.57	566.28	229.80	149.14	499.06	277.66	214.15	105.08	378.90	325.40
2011	143.28	138.44	180.24	98.63	250.37	180.65	97.63	124.41	231.53	72.95	44.07	26.60	115.75	146.35
2012	236.97	237.55	352.53	248.00	454.08	325.00	112.19	219.28	458.53	227.04	220.77	99.89	502.00	297.98
2013	145.72	178.06	229.92	190.28	367.63	267.31	147.06	135.71	433.95	148.91	135.94	84.84	236.11	233.09
2014	156.09	177.79	229.30	274.30	476.01	229.23	260.49	271.40	359.26	218.69	412.16	244.24	515.23	294.06
2015	195.21	234.03	251.82	310.60	516.46	270.90	287.44	324.70	462.70	207.40	548.19	268.06	366.18	335.63
Total	171.07	161.92	241.54	209.49	408.62	274.01	156.15	170.54	339.85	163.54	200.00	114.89	319.06	236.11

**Table 4:** Incidence of HFMD infection per 100,000 by enterovirus serotype

	EV 71	CV A16	UEV	Total
2008	0.10	0.09	0.03	0.22
2009	1.15	0.16	0.10	1.41
2010	1.37	1.65	0.71	3.73
2011	1.50	0.19	0.61	2.30
2012	4.86	1.36	1.46	7.68
2013	1.95	1.59	6.94	10.49
2014	5.62	3.75	1.36	10.73
2015	2.90	2.47	5.69	11.06
Total	2.49	1.44	2.19	6.12

**Table 5:** Disease features of the three serotypes of enterovirus in Xi’an City, China during 2008–2015^a^

	Laboratory-confirmed	Median age, y	Fatalities	Severe
EV-A71	1641 (57.40%)	2.00	15 (0.91%)	710 (43.27%)
CV-A16	719 (25.15%)	2.00	1 (0.14%)	95 (13.21%)
UEV	499 (17.45%)	2.00	0	229 (45.89%)

^a^Reported as case n (%), unless indicated otherwise Zhang et al. [13]

### Temporal distribution of HFMD incidence

**Fig. 2:**
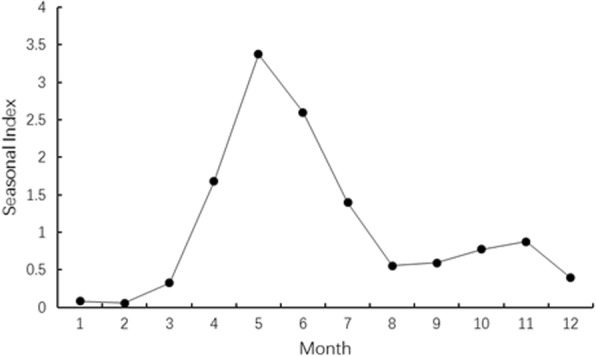
Seasonal variations of HFMD infections during 2008–2015 in Xi’an City, China

The seasonal variations in rate of HFMD infection are shown in Fig. 2. A small peak of HFMD infections (22.54%) occurred during September and November, and a large peak occurred during April and July (66.11%). The secular trend in HFMD infections was calculated based on the estimated annual incidence rate. A fluctuating but increasing temporal trend of incidence rate, together with declining trend of fatality rate due to HFMD were identified (Cochran-Armitage trend test, incidence rate: *Z* = 102.5, *P* < 0.05; mortality rate: *Z* = − 0.2, *P* > 0.05; case fatality rate: *Z* = − 2.3, *P* < 0.05).

### Spatial distribution of HFMD infections

**Fig. 3:**
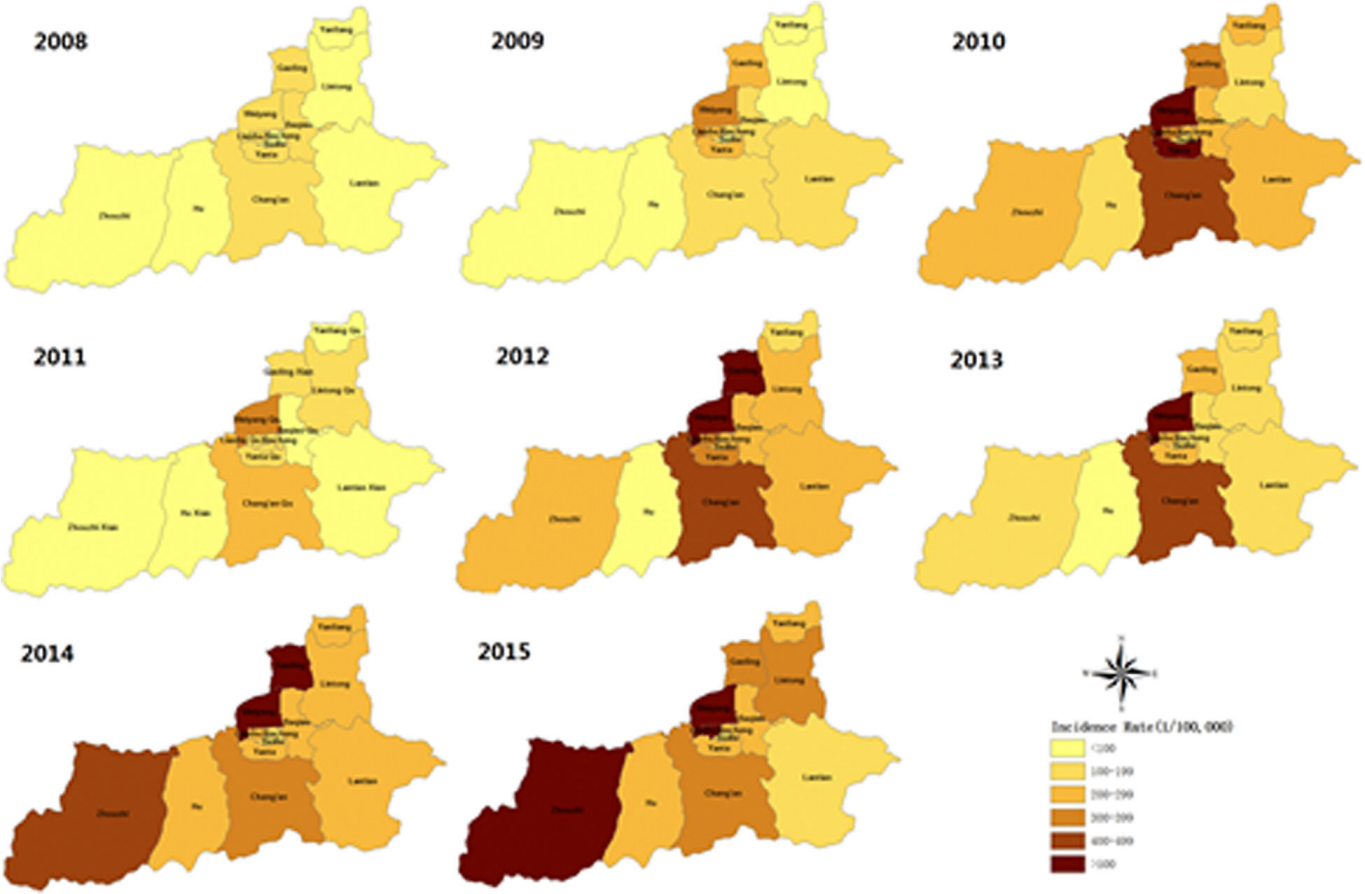
Spatial trend of HFMD incidence based on annual incidence rate at the county level during 2008–2015 in Xi’an City, China

The spatial distribution of HFMD incidence rate based on the annual incidence rate at the county level in Xi’an City during 2008 and 2015 was illustrated in Fig. 3. Weiyang district had the highest average incidence rate during the entire period. The highest incidence rate of HFMD occurred in 2015 in Zhouzhi district (548.19/100,000), while the lowest rate occurred in 2008 in Zhouzhi county (8.21/100,000) (Table 3).

**Fig. 4:**
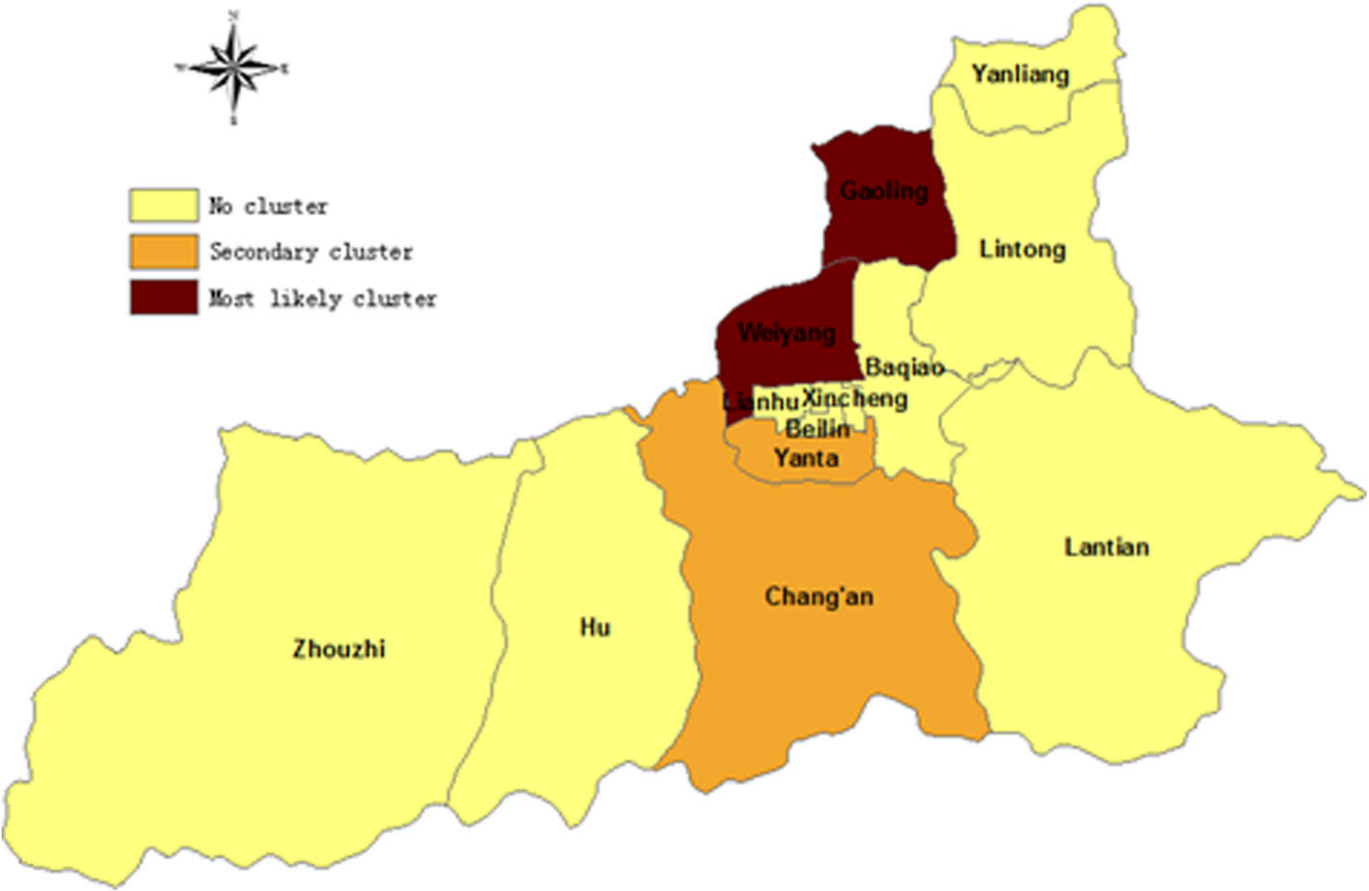
Spatial distribution of clusters with high HFMD risk during 2008–2015 in Xi’an City, China

The spatial cluster analysis of HFMD incidence rate during 2008–2015 showed that Weiyang district and Gaoling county together constitute the cluster with the highest HFMD incidence rate (average annual incidence = 378.17/100,000; RR = 1.81, *P* < 0.01), followed by Yanta district and Chang’an district as the cluster with the second highest HFMD incidence rate (average annual incidence = 305.45/100,000, RR = 1.52, *P* < 0.01; Fig. 4).

### Enterovirus serotype distribution

A total of 2859 HFMD cases were confirmed in the laboratory, of which 1641 (57.40%) cases were infected by EVA71, 719 cases (25.14%) by CVA16, and 499 cases (17.45%) by untyped enteroviruses (UEV) (Table 5).These cases were from the surveillance test program. The incidence rate Incidence of HFMD infection per 100,000 by these three enterovirus serotype through 2008–2015 were 2.49(EV71), 1.44(CVA16), 2.19(UEV) (Table 4).

**Fig. 5:**
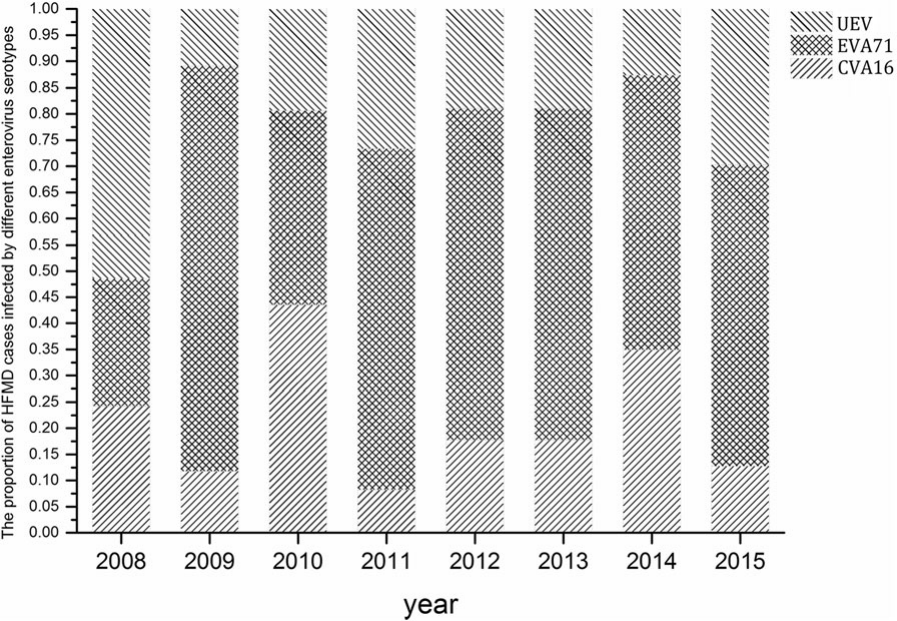
Proportion of HFMD cases infected by 3 enterovirus serotypes during 2008–2015 in Xi’an City, China

The annual proportion of HFMD cases infected by these 3 serotypes was not consistent from 2008 to 2015 (Fig. 5). During 2011 and 2015, EVA71 was the primary attacking enterovirus, accounting for 50.0% of all infection. EVA71 caused highest number of fatalities (0.91%), while UEV caused the most severe cases (45.89%) (Table 5).

## Discussion

The study confirmed that Xi’an city was a major center of HFMD infections after compare the average incidence rate with other capital cities and listed the higher IR cities here such as Beijing (IR = 164.07/100,000, during 2007–2015) [13] Wuhan(IR = 147.45/100,000,during 2011–2016) [14], Taiyuan(IR = 166.64/100,000, during 2012–2014) [15], Shenyang(IR = 116.04/100,000, during 2012–2016) [16], Tianjin(IR = 116.41/100,000, during 2013–2014) [17], and for Xi’an, it has an average annual incidence rate of 235.01 per 100,000 population which is higher than most capital cities in the national wide. Also, the epidemic tendency is consistent with previous reports in China [18, 19], Children, especially boys younger than 5 years, were the most vulnerable group. In Xi’an, the largest seasonal outbreak occurred during April and July, followed by a smaller peak during September and November. We also observed that central regions of Xi’an city were the major area of the HFMD outbreaks. EVA71 was the predominant enterovirus serotype causing HFMD.

Xi’an has a high incidence rate and lower case fatality rate comparing with the national wide. The national incidence of HFMD in China during 2010–2012 was reported 1.2 per 1000 person-years [2]. Our study observed that the estimated incidence rate of HFMD infections in Xi’an city during 2010–2012 was higher than the average national level. Although the incidence rate of HFMD in Xi’an was high with an increasing temporal trend, the case fatality rate was however declining. In May 2008, China established the national surveillance system for HFMD, in order to improve the case- finding ability and reduce the case failed to report. Control and prevention of HFMD.

The highest outbreaks of HFMD in Xi’an City were observed during April through July, followed by September and November, which was consistent with results reported in other areas of China. In Zunyi city, the highest incidence rates of HFMD infection occurred during May–July and October–December [20]. In Guangxi Province, the highest rate occurred during April–July [8]. And in Guangdong Province, the highest seasonal incidences was May–June and October–November [6]. Temperature and climate influence the viral activity [21], and consequently, may be associated with the chance of infection in susceptible populations. These results implicate that control and prevention shall be launched before the anticipated peak of seasonal infections of HFMD in Xi’an.

This study also revealed that the areas of high RR of HFMD were the central regions of Xi’an located around the urban region, specifically Weiyang District, Gaoling County, Yanta District, and Chang’an District. This finding indicates the need to focus HFMD control and prevention measures in central Xi’an, to make the allocation of public health resources most cost-effective and beneficial.

There is one limitation in our study, which is about the dominant etiological pathogens. Before 2012, EVA71 and CVA16 were the predominant etiological pathogens of HFMD among young children in China [1]. In recent years, the pathogens responsible for HFMD infections in China have become more diverse; the percentages of EVA71 and CVA16 infection have decreased, while that of CVA16 has increased in some areas of China [22–25]. But in Xi’an, only the serotypes EVA71 and CVA16 were detected, and other serotypes were defined as untyped enteroviruses (UEV), and our laboratory did not has the reagent to test other serotypes such as CA6 and CA10 which also be the high incidence serotypes in China recent years. This might be insufficient in our study and future study could complement this part.

The present study showed that, although the proportion of cases infected by UEV fluctuated, EVA71 was still the predominant pathogen of HFMD in Xi’an City. It was reported that EVA71-associated HFMD cases are more likely than those of other serotypes to develop potentially fatal neurological and systemic complications [26]. This study also showed that the fatality rate in EVA71-associated HFMD cases was higher than that of other serotype-associated HFMD cases. Therefore, we should focus on control and prevention of EVA71-associated HFMD, and monitor closely the fluctuations of other serotype-associated HFMD infections.

Meanwhile, the first EVA71 vaccine has now launched in China. The vaccine could give more than 90% protection against clinical EVA71-associated HFMD [27, 28], which should assist with the control and prevention of HFMD in Xi’an.

This study also observed that the incidence rate of HFMD infections in Xi’an City was high for boys younger than 5 years, which is consistent with previous reports in other areas of China [2, 7, 20, 29, 30]. Therefore, we should focus HFMD programs for control and prevention on children who are aged less than 5 years, especially boys.

It should also be noted that China established the national surveillance system for HFMD in May 2008. Therefore, the number of HFMD cases and deaths from January to April in 2008 may be under-reported. Another limitation of this study was that only a few samples were tested for enteroviruses, and specific serotypes other than EVA71 and CVA16 were not tested in Xi’an. Studies are needed to monitor the fluctuation in HFMD infections in Xi’an over longer periods, and more samples should be tested to identify comprehensively the serotypes of enteroviruses.

In summary, the current study confirmed that children, especially boys under the age of 5 years, were the group most susceptible to HFMD infection. The seasonal outbreaks in Xi’an occur from April through July and from September through November. We also observed that the central regions of Xi’an city were the major locations of infections. These findings can be helpful for the prevention and control of HFMD infections in the future.

## Conclusion

From the study, we come to the conclusion that HFMD morbidity rate was increasing by years, and mortality rate was decreasing respectively in Xi’an Northwest China. Furthermore, EV71 is the primary attacking entervirus since 2011 and composed 50% of all the infection cases. The result pointed out that season, region and focus groups should be considered priority to control the developments of the epidemics. Children who under 5 years old is the adaptation people to receive vaccination, which means by promoting the vaccine injection, severe cases and death cases will be decreased.

## Abbreviations

CDC: Center for Disease Control and Prevention
CVA16: Coxsackie virus A16
EVA71: Enteroviruses A71
HFMD: Hand-foot-and-mouth disease
PCR: Polymerase Chain Reaction
